# Single cell RNA-seq analysis of the flexor digitorum brevis mouse myofibers

**DOI:** 10.1186/s13395-021-00269-2

**Published:** 2021-05-17

**Authors:** Rohan X. Verma, Suraj Kannan, Brian L. Lin, Katherine M. Fomchenko, Tim O. Nieuwenhuis, Arun H. Patil, Clarisse Lukban, Xiaoping Yang, Karen Fox-Talbot, Matthew N. McCall, Chulan Kwon, David A. Kass, Avi Z. Rosenberg, Marc K. Halushka

**Affiliations:** 1 Department of Pathology, Johns Hopkins University School of Medicine, Ross Bldg. Rm 632B, 720 Rutland Avenue, Baltimore, MD 21205 USA; 2 Division of Cardiology, Department of Medicine, Johns Hopkins University School of Medicine, Baltimore, MD USA; 3 Department of Biostatistics and Computational Biology, University of Rochester Medical Center, Rochester, NY USA

**Keywords:** Single cell RNA-sequencing, Skeletal muscle, Twitch, Fiber

## Abstract

**Background:**

Skeletal muscle myofibers can be separated into functionally distinct cell types that differ in gene and protein expression. Current single cell expression data is generally based upon single nucleus RNA, rather than whole myofiber material. We examined if a whole-cell flow sorting approach could be applied to perform single cell RNA-seq (scRNA-seq) in a single muscle type.

**Methods:**

We performed deep, whole cell, scRNA-seq on intact and fragmented skeletal myofibers from the mouse fast-twitch flexor digitorum brevis muscle utilizing a flow-gated method of large cell isolation. We performed deep sequencing of 763 intact and fragmented myofibers.

**Results:**

Quality control metrics across the different gates indicated only 171 of these cells were optimal, with a median read count of 239,252 and an average of 12,098 transcripts per cell. scRNA-seq identified three clusters of myofibers (a slow/fast 2A cluster and two fast 2X clusters). Comparison to a public skeletal nuclear RNA-seq dataset demonstrated a diversity in transcript abundance by method. RISH validated multiple genes across fast and slow twitch skeletal muscle types.

**Conclusion:**

This study introduces and validates a method to isolate intact skeletal muscle myofibers to generate deep expression patterns and expands the known repertoire of fiber-type-specific genes.

**Supplementary Information:**

The online version contains supplementary material available at 10.1186/s13395-021-00269-2.

## Background

Skeletal muscle is a voluntary, striated muscle found throughout the body with contraction regulated by nerve impulses through the neuromuscular junction (NMJ). Skeletal muscles consist of different fiber types delineated by the isoform of the myosin heavy chain they express, metabolic function, and other properties [1]. Mouse skeletal muscles are comprised of slow fibers (type 1) and three types of fast fibers: type 2A, type 2B, and type 2X [2–4]. These fiber types are variable across different muscles of the body reflecting different functional needs [2, 4]. Our understanding of all of the genes that vary across these fiber types is limited, although many well-characterized examples such as myosin heavy chains, calcium ATPase pumps, and metabolic proteins are known. Only recently has there been an effort to catalog the entirety of fast-/slow-twitch expression differences by single cell approaches.

The most comprehensive gene expression study was performed in mice using DNA microarrays across ten type 1 and ten type 2B fibers [5]. Single cell RNA-sequencing (scRNA-seq) also has been performed in skeletal muscle and muscle cultures. However, until quite recently, the large size of skeletal myofibers has precluded them from these datasets, which are instead predominately satellite cells and other supporting cell types [6–13]. A recent publication used SMART-Seq to evaluate three fast fiber mouse fibers [14], and several single nucleus RNA-seq (nuc-seq) projects have also added to the literature [15–17]. The totality of these studies strongly suggests there are numerous expression differences between skeletal muscle fiber types and demonstrates a need for new approaches to capture this diversity.

The Kwon lab recently established a protocol for scRNA-seq of large mature cardiac myocytes through large particle fluorescence-activated cell sorting (FACS) [18]. We ascertained if this method could be used to isolate the even larger skeletal muscle myofibers for scRNA-seq, as typically only small cells are captured in traditional scRNA-seq methods [6, 8]. We utilized the flexor digitorum brevis (FDB), a well-characterized fast-twitch fiber muscle of the base of the foot, made up predominately of type 2A (IIa) and type 2X (IIx) fibers [19]. Our goal was to validate this whole cell capture method, compare whole cell single cell data to single nuclear data, and characterize this important model muscle.

## Methods

### Isolation and sequencing of adult skeletal myofibers

All animal studies were approved by the Institutional Animal Care and Use Committee at Johns Hopkins and all methods were performed in accordance with the relevant guidelines and regulations. This study used adult male mice (>3 months) from the C57BL/6J and DBA2 backgrounds (Jackson Labs, Bar Harbor, ME). All mice were first anesthetized in an induction chamber using isoflurane until breathing rate has slowed to 1 Hz and were unresponsive to rear toe pinches. This was followed by cervical dislocation prior to excision of any muscles. To isolate skeletal myofibers, we performed collagenase-based digestion of the flexor digitorum brevis (FDB), a short muscle of the hind feet, as per previously established protocols [20]. We performed intact and fragmented FDB studies. The FDB was transferred to a dish containing DMEM with 1% penicillin/streptomycin, 1% fetal bovine serum, and 2mg/mL Collagenase Type II (Worthington). Muscle was digested for 1.5 h in a 37°C cell incubator with 5% CO_2_. Subsequently, the muscle was transferred to a dish containing media without collagenase and gently triturated to release single myofibers. Large undigested chunks and tendons were removed with tweezers prior to single cell isolation. A COPAS SELECT Flow Pilot Platform (Union Biometrica) was employed, as described below.

These sorted cells were placed individually into 96-well plates. Capture plate wells contained 5 μl of capture solution (1:500 Phusion High-Fidelity Reaction Buffer, New England Biolabs; 1:250 RnaseOUT Ribonuclease Inhibitor, Invitrogen). Single cell libraries were then prepared using the previously described mcSCRB-seq protocol [21, 22]. Briefly, cells were subjected to proteinase K treatment followed by RNA desiccation to reduce the reaction volume. RNA was subsequently reverse transcribed using a custom template-switching primer as well as a barcoded adapter primer. The customized mcSCRB-seq barcode primers contain a unique 6 base pair cell-specific barcode as well as a 10-base pair unique molecular identifier (UMI). Transcribed products were pooled and concentrated, with unincorporated barcode primers subsequently digested using Exonuclease I treatment. cDNA was PCR-amplified using Terra PCR Direct Polymerase (Takara Bio). Final libraries were prepared using 1ng of cDNA per library with the Nextera XT kit (Illumina) using a custom P5 primer as previously described.

### scRNA-seq sequencing and analysis

Pooled libraries were sequenced on two high-output lanes of the Illumina NextSeq500 with a 16-base pair barcode read, 8-base pair i7 index read, and a 66-base pair cDNA read design. To analyze sequencing data, reads were mapped and counted using zUMIs 2.2.3 with default settings and barcodes provided as a list [23]. zUMIs utilizes STAR (2.5.4b) [24] to map reads to an input reference genome and featureCounts through Rsubread (1.28.1) to tabulate counts and UMI tables [24, 25]. Reads were mapped to the mm10 version of the mouse genome. We used GRCm38 from Ensembl concatenated with ERCC spike-in references for the reference genome and gene annotations. Dimensionality reduction and cluster analysis were performed with Seurat (2.3.4) [26].

### Seurat-based analysis

Analysis was performed using the Seurat R toolkit V3.1.1 for this dataset [26]. Initial filtering removed lower quality cells (read count <5000 RNAs detected or >20% mitochondrial genes) before sctransform normalization [27]. We performed principal components analysis (PCA) of the top 3000 variable genes based on the Seurat sctransform algorithm and used the top 4 for downstream analysis. We generated a Seurat workflow that identifies a subset of genes with high cell-to-cell variation within the scRNA-seq data. A Uniform Manifold Approximation and Projection (UMAP) was generated alongside a heat map representing the top genes in clusters as determined by each gene set used for PCA.

### Analysis of a public nuclear RNA-Seq dataset

The snRNA analysis was done in Seurat V3.1.1 taking data available from Dos Santos et al. [28] https://www.ncbi.nlm.nih.gov/geo/query/acc.cgi?acc=GSE150065. Their data consisted of four sets of matrices, one of which was a mix of tibialis, extensor digitorum longus, gastrocnemius, and plantaris, which we refer to as “mixed muscle” [28]. The other three sets were separate quadriceps, tibialis, and soleus. We retained nuclei from all muscle samples in mixed muscle that contained 200–2500 unique RNAs and had less than 5% mitochondrial genes. Log normalization was performed before finding the top 2000 variable features and scaling through Seurat’s built in functions. These variable features were used to build principal components and the top 10 were used for clustering and UMAP visualization. We then subselected myocyte nuclei using *Ttn* as a positive marker and *Abca8a* and *Plxdc2* as negative markers of fibroadipogenic progenitors. We painted the UMAP of the remaining nuclei using *Myh1*, *Myh2*, *Myh4*, and *Myh7* to identify muscle fiber types. Seurat’s Findallmarkers used a Wilcoxon rank-sum test to identify differentially expressed genes between clusters expressed in at least 50% of each cluster being examined. We then used these genes and myosin heavy chains to assign identities to slow, fast2A, fast 2B, and fast 2X clusters.

Finally, we ran differential gene expression analysis using a *T*-test between the Fast 2X and a combined Slow/Fast2A group, to match the analysis of the prior scRNA-seq data. A logFC threshold of 0.35 filtered out highly abundant genes.

### RISH

Wild-type C57Bl6 mouse skeletal muscles (extensor digitorum longus, gastrocnemius, soleus, diaphragm) and brain were obtained at necropsy under an approved ACUC protocol. Tissues were immediately fixed in formalin and paraffin-embedded blocks were created, from which 5-μm slides were made. Catalog probes for RNA in situ hybridization (RISH) were obtained from RNAscope (ACDBio). These probes were designed to detect the following genes: *Myh2* (pre-mRNA, #539031-C2), *Got2* (#459111), *Fhl1* (#536521), *Ntrk3* (423621-C2), and *Gabbr2* (#317971). Each probe set targeted all validated NCBI refseq transcript variants of the gene. One custom probe, *Eno3*, was designed to target all transcript variants of *Eno3* (GeneBank accession nm_007933.3).

The Multiplex Fluorescent Reagent Kit v2 (ACDBio) was used following the manufacturer’s instructions. Briefly, FFPE tissue slides were baked for 1 h at 60°C. The slides were subsequently deparaffinized with xylene, rinsed with 100% ethanol and air-dried. After application of hydrogen peroxide and washing, slides were treated with the target retrieval reagent in a steamer (>99°C) for 20 min. Then, the tissue was permeabilized using a protease. Hybridization of the probes to the targeted mRNAs was performed by incubation in a 40°C oven for 2 h. After washes, the slides were processed for the standard signal amplification and application of fluorescent dye (Opal dyes 520 and 570, AKOYA Biosciences) steps. Finally, the slides were counterstained with DAPI, mounted with Prolong Gold Antifade Mounting solution (Invitrogen), and stored in a 4°C room. The fluorescent images were obtained in the Johns Hopkins Microscope Core Facility using a Zeiss LSM700 Laser scanning confocal microscope. Images were manually counted for co-expression, counter-expression, and non-expression across muscle fibers in ImageJ [29], and a *χ*^2^ analysis, with Yates correction, was determined in Rstudio (v1.3.1093) and R (v4.0.3).

### Human Protein Atlas

The HPA is a comprehensive repository of IHC-stained tissue microarrays for numerous tissues, including skeletal muscle [30, 31]. We cross-referenced our gene list with the HPA to find examples of concordance and discrepancy to our gene list for variable expression.

### Gene Ontology (GO) validation

GO was performed on the 557 most variable genes between two fast 2X clusters (2X_c1_ and 2X_c2_) using the Gene Ontology resource (http://geneontology.org/) and selecting for the cellular component. Gene lists for terms “actin cytoskeleton,” “mitochondria,” “cell-cycle,” and “transcription regulator activity” were obtained from GO and used to determine the average expression of genes in each category from the single cell RNA-seq and nuc-seq datasets. The log2 normalized expression values of the datasets were normalized to each other.

### Data availability

Mouse skeletal muscle sequencing was deposited at the Sequence Read Archive (SRA – SRP241908) and the Gene Expression Omnibus (GSE143636).

### Code availability

All analysis scripts are available at GitHub (https://github.com/mhalushka/Skeletal_muscle_mosaicism).

## Results

### Validation of a large cell scRNA-seq method

**Fig. 1:**
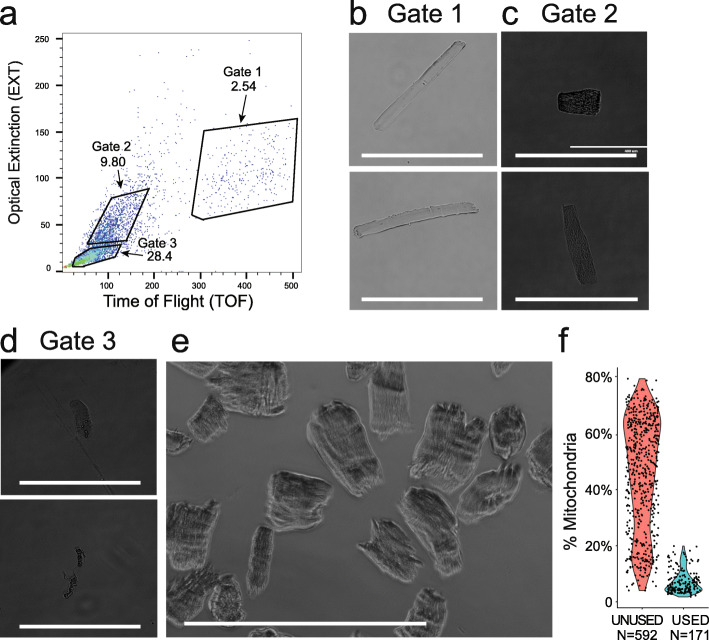
FDB muscle myocyte preparation. **a** Flow cytometry showing three gated areas representing EXT-high/TOF-high, EXT-high/TOF-low and EXT-low populations of FDB myofibers. **b** Representative images of Gate 1 EXT-high/TOF-high. **c** Representative images of Gate 2 EXT-high/TOF-low. **d** Representative images of Gate 3 EXT-low. **e** Representative image of pseudo-biopsy isolated myocyte fragments. Gates 1 and 2 were used for library preparation. White size bar is 400 μm. **f** Percent of mitochondria in unused and used cells

We utilized a large particle FACS (LP-FACS) method testing two different approaches to isolated and dissociated FDB myofibers. In the first approach, we dissected the FDB from tendon to tendon prior to digestion, enabling isolation of fully intact myofibers. In the second approach, we cut small portions of the FDB muscle using scissors. We reasoned that the latter approach would broadly mimic skeletal muscle needle biopsies as might be done, for example, from a human patient sample. We isolated single myofibers through LP-FACS, using a flow channel size of 500 μm. The COPAS SELECT Flow Pilot Platform was employed. Using time-of-flight (TOF, measuring axial length) and optical extinction (EXT, measuring optical density) parameters, we found that skeletal myofibers separated into three populations—an EXT-low population, EXT-high/TOF-low population, and EXT-high/TOF-high population (Fig. 1a). The EXT-high/TOF-high population was comprised almost entirely of intact myofibers with lengths > 400 μm, suggesting successful sorting of large myofibers (Fig. 1b). Interestingly, the EXT-high/TOF-low population was composed of what appeared to be rod-shaped fragments that maintained sarcomeric proteins, albeit disrupted (Fig. 1c). The EXT-low population was comprised mostly of debris and dead cells, as previously observed with cardiac myocytes (Fig. 1d). The EXT-high/TOF-low population qualitatively resembled our pseudo-biopsy isolated myofiber fragments (Fig. 1e), which also shared similar TOF and EXT parameters (not shown). To our knowledge, this is the first FACS-based single cell RNA-seq study of skeletal myofibers; thus, we adopted a broad gating strategy for isolation of single cells. We sorted 700 EXT-high myofibers (comprised of both TOF-high and TOF-low populations) as well as 100 myofiber fragments isolated through the pseudo-biopsy method.

The two gates and pseudo-biopsy approach were used to isolate 763 cells for single cell RNA-seq using the established mcSCRB-seq protocol [21, 22]. The entire group of 763 cells/cell fragments were sequenced to a median depth of 108,110 reads per cell. Preliminary analyses, however, indicated a distinct cluster of cells with a high percentage of mitochondrial reads (Fig. 1f) or otherwise low abundance reads (median 12,187 per cell). Notably, almost all of our pseudo-biopsy myofiber fragments and many TOF-low cells fell into this category. These quality control metrics likely indicated poor quality or sheared cells with loss of RNA. Thus, we excluded these cells, identified the EXT-high and TOF-high gate as the appropriate gate to obtain high quality myofibers, and narrowed our analysis to the best 171 cells (>5000 genes expressed and <20% mitochondrial genes) remaining with a median read count of 239,572 per cell.

### Analysis of the expression patterns of single FDB myofibers

A median of 12,187 transcripts were identified in these myofibers and all had the expression patterns of mature skeletal myofibers, highly expressing a myosin heavy chain isoform.

**Fig. 2:**
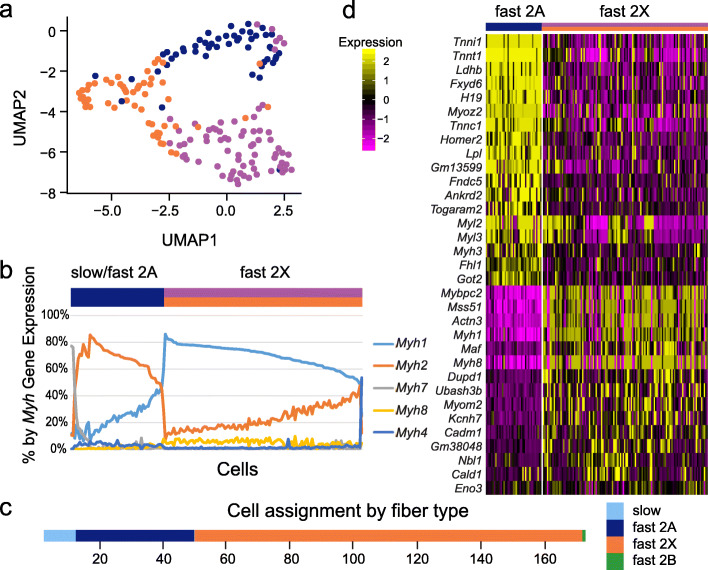
Subtyping of skeletal myofibers. **a** UMAP graph of 171 skeletal muscle cells based on variable gene expression, indicating 3 clusters. **b** Major myosin heavy chain distributions across the 171 cells as a percentage of each heavy chain. **c** Assignment of each cell to a fiber type. **d** Heat map of major gene expression differences between fast 2A and fast 2X cells

We used the top 4 significant PCs to cluster these cell types (Fig. 2a). Three groups were observed in a UMAP dimensionality reduction plot. Two clusters, containing 69 and 53 cells respectively (71% of all cells) had elevated expression of *Myh1* and *Myh8* identifying these groups as fast 2X type cells. *MYH8*, while considered a neonatal myosin, maintains low expression in adult skeletal muscle [32, 33]. A third cluster containing 49 cells was defined by high expression of *Tnnt1* and *Myh2.* A deeper analysis of this group showed that 12 cells had high to modestly elevated *Myh7* expression (a slow-twitch marker), indicating this cluster was a combination of slow-fibers cells and fast 2A fibers (Fig. 2b, c). Of note, *Myh4*, a myosin heavy chain associated with fiber type 2B, was the dominant myosin in only a single cell that was assigned to this group (Fig. 2b, c) [2]. As the FDB is a fast-fiber muscle, the overall distribution of significantly more fast (159) to slow fibers (12) is consistent with expected.

Interestingly, the expression patterns of the main fast-/slow-fiber differentiating *Myh* genes was not as dichotomous as noted in protein based fiber type data [34]. Here there were many more cells with intermediate levels and coexpression of *Myh1* and *Myh2* suggesting higher gene plasticity and more cell hybrids (Fig. 2b) [2].

### Shared and variable transcripts by cell type

We wondered about the extent to which highly abundant genes were mosaic across these cell fiber states. By normalized read counts of the scRNA-seq data, we determined the 50 most abundant transcripts by the average of each cell type in the two fast 2X clusters and the one fast 2A / slow cluster determined by Seurat (Suppl. Table 1). The overall most abundant transcripts were *Ttn*, *Acta1*, and *mt-Rnr2*. There was significant overlap of abundant genes, with only 9 genes being different across the three samples. We then explored differences specifically between the two most abundant fiber types, fast 2A and fast 2X. Of 2649 evaluated genes (all expressed in ≥95% of cells of one cluster), 160 genes were differentially expressed across the two groups (*t*-test, adj. *p* value <0.01). This included expected genes such as *Tnni1*, *Tnnt1*, and *Myh1* and less investigated genes such as *Ubash3b* and *Togaram2* (Fig. 2d, Suppl. Table 2).

**Fig. 3:**
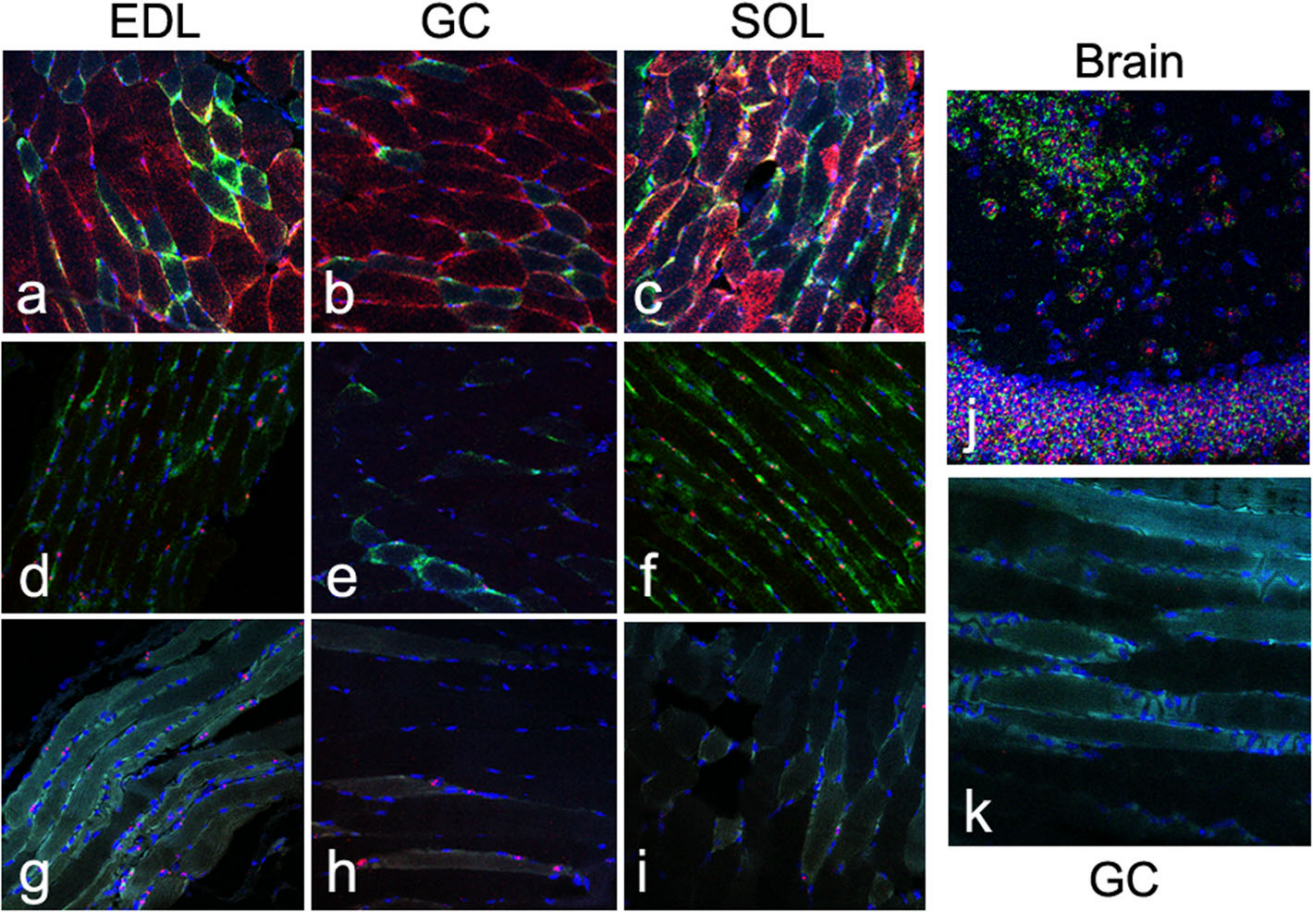
RISH staining of variably expressed genes across extensor digitorum longus (EDL), gastrocnemius (GC), and soleus (SOL). **a**–**c** *Fhl1* (green) and *Eno3* (red) show differing expression patterns across EDL (**a**), GC (**b**), and SOL (**c**). There is reduced *Fhl1* in the fast-twitch GC and increased *Fhl1* in the slow-twitch SOL fiber. **d**–**f** *Fhl1* (green) is coexpressed with *Myh2* (red), which has a perinuclear pattern. Both *Fhl1* and *Myh2* are reduced in GC (**e**) and increased in SOL (**f**). **g**–**i** *Got2* (green) is coexpressed with *Myh2* (red) showing highest expression in the EDL (**g**). **j** Neuronal tissue showing strong staining of *Gabbr2* and *Ntrk3*. **k** *Gabbr2* shows a variable blush across the GC, while no discernable *Ntrk3* was observed in the GC. Nuclei were stained with DAPI (blue) in all images

We validated a subset of these genes (*Eno3*, *Fhl1*, *Got2*, *Myh2*) using RISH and available probes across the extensor digitorum longus (EDL), gastrocnemius, and soleus. *Eno3* is a known fast fiber gene (both 2A and 2X) and was identified in most cells of the fast-twitch EDL and gastrocnemius (Fig. 3a, b). *Fhl1* was identified as being elevated in fast 2A myofibers (Fig. 2d, Suppl. Table 2). In *Fhl1*-positive myofibers, *Eno3* qualitative expression was reduced, but not absent. In the slow-twitch soleus (Fig. 3c), levels of both genes were decreased. Of 693 myofibers reviewed across all of the tissues, most (341, 49%) showed co-expression, with 186 cells being *Eno3*+ only, 92 being *Fhl1*+ only and 74 having no expression. A *χ*^*2*^ analysis demonstrated only a modest enrichment for co-expression (*χ*^2^=4.25, *p* = 0.039). *Fhl1* was then compared to *Myh2*, a known fast 2A gene (Fig. 3d–f). The strong pre-mRNA *Myh2* staining was interpreted as nuclear [15, 16]. The expression of the two genes demonstrated appropriate overlap in the same cells (206 co-expressed, 195 non-expressed, and 30 counter-expressed, *χ*^2^ = 320.9, *p* = 9.3e-72). *Got2*, also identified as elevated in fast 2A fibers, showed appropriate co-expression with *Myh2* across all three tissues (Fig. 3g–i) and by myofiber (81 co-expressed, 69 non-expressed, 22 counter-expressed, *χ*^2^ = 98.1, p = 4.0e−23). These patterns of fast fiber expression are consistent with those identified by scRNA-seq (Fig. 2d).

### Comparison of full cell scRNA-Seq to nuclear RNA-Seq

A recent publication by Dos Santos et al. [16] described nuc-seq of mouse skeletal muscles from a mixed sample of tibialis, gastrocnemius, soleus, plantaris, and extensor digitorum longus (*N*=6 each), along with nuclei from each of quadriceps, tibialis, and soleus, identifying myonuclei based on *Ttn* expression. Whereas we focused on sequencing depth (239,572 median reads/171 cells), Dos Santos et al. went wide, obtaining many more skeletal myofiber nuclei (6962), but only to a median read count of 2785 and 1210 transcripts per nucleus in their mixed muscle sample. We processed this dataset using Seurat and determined, as they reported, the presence of slow, fast 2A, fast2A/2X, fast 2B, and fast 2X nuclei clustering more distinctly by myosin heavy chain status on a UMAP visualization of the data, than our whole scRNA-seq data (Suppl. Fig. 1).

As the whole cell versus nuclear isolation methods were so distinct, we evaluated how those differences affect the presence of abundant genes. Notably, in a comparison of the most highly expressed genes, only 27 were present in the top 100 for both methods. A GO search of the 73 genes that were only abundant in the whole cell scRNA-seq showed these genes were enriched for terms such as “myofibril” and “ATP metabolic process.” This had us wonder if we could observe differences in gene classes based on the nuc-seq vs scRNA-seq methods similar to that described in other cell types [35]. We used normalized expression data between the studies and determined the expression differences between whole cell and nuclear data for the genes representing the GO terms of transcription factors, cell cycle genes, mitochondria, and actin-cytoskeleton. Both transcription factors and cell cycle genes were more abundant in the nuc-seq data (3.9 and 1.9 fold respectively), while actin-cytoskeleton genes were more abundant in scRNA-seq data (1.9 fold). Mitochondrial genes were equivalent for expression across the two methods. However, two of the most abundant scRNA-seq genes overall, the mitochondrial genes *mt-Rnr1* and *mt-Rnr2*, were not present in the nuc-seq data, impacting these results. Also of note, *Malat1*, a known nuclear lncRNA, was the most abundant transcript in the nuc-seq data, consistent with a prior report [35].

We compared a combined slow/fast 2A group against a fast 2X group to again discern differences in myofiber-specific genes by sequencing method. There were 771 differentially expressed genes (*t*-test, adj. *p* value <0.01) in comparison to 152 in the whole cell dataset. Only 80 differentially expressed genes overlapped between the two datasets and of these, three were significant in opposite fold directions (*Crim1*, *Myh4*, *Kcnc1*) (Suppl. Table 3). Altogether, these data indicate the ability to discern cell myofiber types, by either nuc-seq or scRNA-seq, despite differences in the specific genes that discriminate across the slow/fast 2A and fast 2X cells and the relative expression levels of genes, by the two methods of RNA-seq.

### Is there a meaningful difference between fast 2X subclusters?

The initial Seurat analysis subsetted the fast 2X cluster into two groups. We explored if these two fast 2X clusters (cluster 1 - 2X_c1_; cluster 2 – 2X_c2_) represent unique cell types, cell states, or some technical division. Of 5260 genes compared, 557 genes were differentially expressed (*t* test; adj. *p* value <0.01). A GO analysis on the 557 genes identified an enrichment of the cellular component “neuronal synapse,” suggesting variability at the NMJ. A further review of the top significant genes showed that >20 genes appear to have neuronal origins (*Cdh4*, *Cdkl5*, *Cntn4*, *Dscam*, *Gabbr2*, *Kirrel3*, *Lingo2*, *Lrp1*, *L1cam*, *Nrcam*, *Ntn1*, *Ntrk3*, *Ptprt*, *Ptpro*, *Robo2*, *Sdk1*, *Sema5a*, *Sema6d*, *Shank2*, *Sox5*, *Tnr*, and *Wwox*). We attempted to exclude technical reasons for this variability before investigating a biological rationale for the division.

First, we noted that the vast majority of the neuronal genes (20/22) were present in at least 120 of the 171 cells (Suppl Fig. 2).We surmised that some degree of ambient RNA was present [36]. We then performed RISH for two of these genes, *Gabbr2* and *Ntrk3*, showing robust neuronal staining (Fig. 3j) and some *Gabbr2*, but no *Ntrk3* in myofibers (Fig. 3k). We further noted *Pecam1* and *Smtn*, as markers of endothelial cells and smooth muscle cells respectively, showed comparable increases in these genes among the fast 2X_c2_ cells. These data indicated that despite extremely low expression, ambient genes, in general, have slightly elevated values in fast 2X_c2_ cells, perhaps consistent with more genes being detected in these cells. We further note that three of the most abundant genes, *Ttn*, *mt-Rnr1*, and *mt-Rnr2* are conversely lower in fast 2X_c2_ cells (Suppl Fig. 2). We take the summation of this data to indicate that the separation of fast 2X_c1_ and 2X_c2_ is a technical artifact related to the sequencing and not a true biological distinction.

## Discussion

Our study represents the first use of LP-FACS to isolate single myofibers for scRNA-seq. This study was designed to prove feasibility of the method and did not attempt to discern fiber type expression across a range of muscle bundles or types, which will be the basis of future studies. Skeletal myofibers are often long, stretching across the length of a muscle; thus, isolation techniques (particularly from human samples) may rely on the use of biopsies or otherwise fragmented myofibers. To test the effect of myofiber fragmentation on scRNA-seq data quality, we used a liberal gating strategy of our dissociated myofibers (including both EXT-high/TOF-low and EXT-high/TOF-high populations) as well as directly sequencing fragmented myofibers generated through a pseudo-biopsy approach. Disappointingly, we found that a large portion of our sequenced myofibers were of poor quality, including those from our pseudo-biopsy approach. By contrast, the highest quality data came from fully intact myofibers, in particular the EXT-high/TOF-high population. Because this population is almost completely enriched for intact myofibers, we believe that future experiments using LP-FACS to isolate skeletal myofibers should focus solely on the EXT-high/TOF-high population. We are confident that this will allow for a much higher percentage of good quality scRNA-seq libraries, akin to what we have observed previously with LP-FACS isolation of cardiac myocytes [18]. These results also mean that more work must be done to identify better isolation methods for human skeletal muscle. Current methods of human skeletal muscle biopsying from the quadriceps only obtain muscle fragments. Although different collection reagents for these biopsies (high potassium and EGTA), which are used to prevent contractions, are used, it remains to be determined if preventing contractions is sufficient to reduce RNA loss as cellular integrity is always lost [37]. If not, more creative means to obtain full length fibers or non-damaged fibers must be considered, including rapid autopsy protocols or larger surgical resections that include skeletal muscles. Otherwise, human muscle data will have to be obtained from nuclei material, which we noted had different, albeit complimentary, expression characteristics.

The recent availability of public nuc-seq skeletal myofiber data allowed us to compare these two techniques. As has been reported, we found significant differences in the gene composition of these cells that was dependent on the sequencing approach [38, 39]. *Myh* gene expression was more distinct in the nuc-seq data. This could be related to technical differences such as a sparser data matrix of nuc-seq with more binary patterns. More interestingly, it could be a biological finding if dynamic transcriptional activity is more distinct in nuc-seq data. The nuc-seq was enriched for transcription factors and cell cycle RNAs, while the whole cell scRNA-seq was biased towards other RNA types including significant expression of the mitochondrial RNAs *mt-Rnr1* and *mt-Rnr2* even after controlling for the percent of mitochondrial RNA. Also, the genes that were variable between the slow/fast 2A and fast 2X populations, across the methods, were frequently inconsistent. Nonetheless, both methods successfully separated myofibers by fast/slow type. It will take additional orthogonal approaches such as proteomics to definitively solve this question of which genes/proteins have variable expression between myofiber types and at what expression levels.

Characterization of the FDB identified essentially two clusters, a fast 2X cluster and a fast 2A/slow fiber cluster. If more slow fibers were sequenced, that second group would have likely separated further. We were able to use RISH to validate some of the genes that had expression differences between the fast 2X and 2A cells. Although our analysis using Seurat subdivided the fast 2X cluster, we believe the simplest explanation of this splitting is a technical cause related to slight differences in very low levels of ambient RNA. A more interesting explanation is variable neuronal transfer of mRNAs across the NMJ into the skeletal muscles via extracellular vesicles [40, 41]. This would imply a real state-difference in these cells, but again is considered unlikely. We feel this exercise in considering technical causes of Seurat-derived cell types is a useful reminder to groups working in the field of defining novel cell types to consider more mundane reasons for some divisions.

In conclusion, we introduce a method of whole skeletal muscle cell isolation for scRNA-seq experimentation. This FDB data is some of the first whole, single cell skeletal myofiber data mainly identifying expression patterns in fast 2A and fast 2X myofibers. Future studies can investigate a variety of muscle beds incorporating more slow or fast 2B cells by this approach.

## Supplementary Information

**Additional file 1 Supplementary Table 1.** sctransformed mean expression values of the 50 most abundant genes by skeletal muscle fiber type.

**Additional file 2 Supplementary Table 2.** Comparison of fast2A and fast2X differential expression.

**Additional file 3 Supplementary Table 3**. Significantly variable genes between fast2A/slow genes and fast2X genes in both the whole cell scRNA-seq and nuc-seq datasets. Average log fold change and adjusted p. value is provided for both scRNA-seq and nuc-seq samples.

**Additional file 4 Supplementary Figure 1.** Myofiber nuclei analysis. **a** A UMAP of all myofiber nuclei with fiber type noted. **b** Myosin heavy chain expression used to assign the myofiber types. **c** Myosin heavy chain expression and number of nuclei by fiber type demonstrating overlapping expression of *Myh* genes in some fiber types. **Supplementary Figure 2.** Comparison of the Fast 2X_c1_ and Fast 2X_c2_ subsets. **a** Twenty-two neuronal or NMJ-related genes are detected in most cells, but enriched in Fast 2X_c2_ (middle left) cells. **b** Three neural genes (*Cdh4*, *Kirrel3*, *Ntn1*), an endothelial specific gene (*Pecam1*) and a smooth muscle cell gene (*Smtn*) are all increased in Fast 2X_c2_ subsets suggesting overall increase of ambient RNA in these cells. **c** Total gene counts are elevated in Fast2X_c2_ subsets despite no increase in total reads or % mitochondria. **d** Typically abundant genes, *Ttn*, *mt-Rnr1*, and *mt-Rnr2* are all of lower expression in Fast 2X_c2_ cells.

## Data Availability

The dataset supporting the conclusions of this article is available in the Sequence Read Archive repository (SRP241908, https://www.ncbi.nlm.nih.gov/sra/?term=SRP241908) and the Gene Expression Omnibus (GSE143636, https://www.ncbi.nlm.nih.gov/gds/?term=GSE143636). The analysis scripts supporting the conclusions of this article are available at the GitHub repository, (https://github.com/mhalushka/Skeletal_muscle_mosaicism).

## Abbreviations

scRNA-seq: Single cell RNA-seq
NMJ: Neuromuscular junction
FACS: Fluorescence-activated cell sorting
FDB: Flexor digitorum brevis
TOF: Time-of-flight
EXT: Optical extinction
UMI: Unique molecular identifier
PC: Principal components
PCA: Principal components analysis
UMAP: Uniform Manifold Approximation and Projection
GO: Gene Ontology
LP-FACS: Large particle FACS
HPA: Human Protein Atlas
