# Supplementary material for: Single cell RNA-seq analysis of the flexor digitorum brevis mouse myofibers
Source: Skelet Muscle. 2021 May 17;11:13. doi: 10.1186/s13395-021-00269-2 (PMC8127317; doi:10.1186/s13395-021-00269-2)
Supplement: Supplementary file 4 — Additional file 4 Supplementary Figure 1. Myofiber nuclei analysis. a A UMAP of all myofiber nuclei with fiber type noted. b Myosin heavy chain expression used to assign the myofiber types. c Myosin heavy chain expression and number of nuclei by fiber type demonstrating overlapping expression of Myh genes in some fiber types. Supplementary Figure 2. Comparison of the Fast 2Xc1 and Fast 2Xc2 subsets. a Twenty-two neuronal or NMJ-related genes are detected in most cells, but enriched in Fast 2Xc2 (middle left) cells. b Three neural genes (Cdh4, Kirrel3, Ntn1), an endothelial specific gene (Pecam1) and a smooth muscle cell gene (Smtn) are all increased in Fast 2Xc2 subsets suggesting overall increase of ambient RNA in these cells. c Total gene counts are elevated in Fast2Xc2 subsets despite no increase in total reads or % mitochondria. d Typically abundant genes, Ttn, mt-Rnr1, and mt-Rnr2 are all of lower expression in Fast 2Xc2 cells. [file 13395_2021_269_MOESM4_ESM.pdf]

Single cell RNA-seq analysis of the flexor digitorum brevis mouse myofibers

Rohan X. Verma[1], Suraj Kannan[2], Brian L. Lin[2], Katherine M. Fomchenko[1], Tim O. Nieuwenhuis[1], Arun H. Patil[1], Clarisse Lukban[2], Xiaoping Yang[1], Karen Fox-Talbot[1], Matthew N. McCall[3], Chulan Kwon[2], David A. Kass[2], Avi Z. Rosenberg[1], and Marc K. Halushka[1]*

1 Department of Pathology, Johns Hopkins University School of Medicine, Baltimore, MD, USA
2 Division of Cardiology, Department of Medicine, Johns Hopkins University School of Medicine, Baltimore, MD, USA
3 Department of Biostatistics and Computational Biology, University of Rochester Medical Center, Rochester, NY, USA

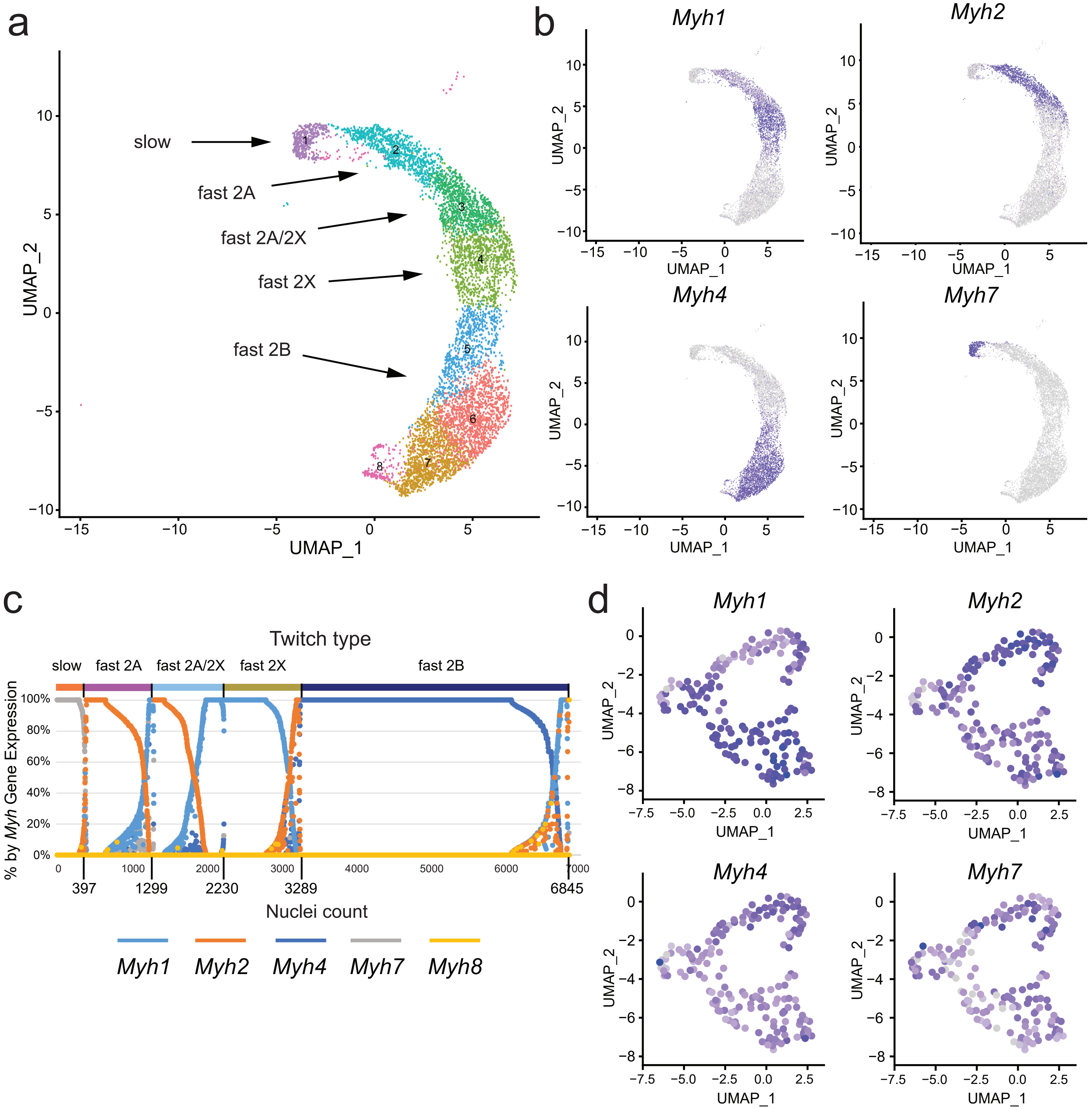

Supplementary Figure S1. Myofiber nuclei analysis. (**a**) A UMAP of all myofiber nuclei with fiber type noted. (**b**) Myosin heavy chain expression used to assign the myofiber types. (**c**) Myosin heavy chain expression and number of nuclei by twitch type demonstrating overlapping expression of Myh genes in some twitch types. (**d**) Myosin heavy chain expression from the whole scRNA-seq data, as a comparison.

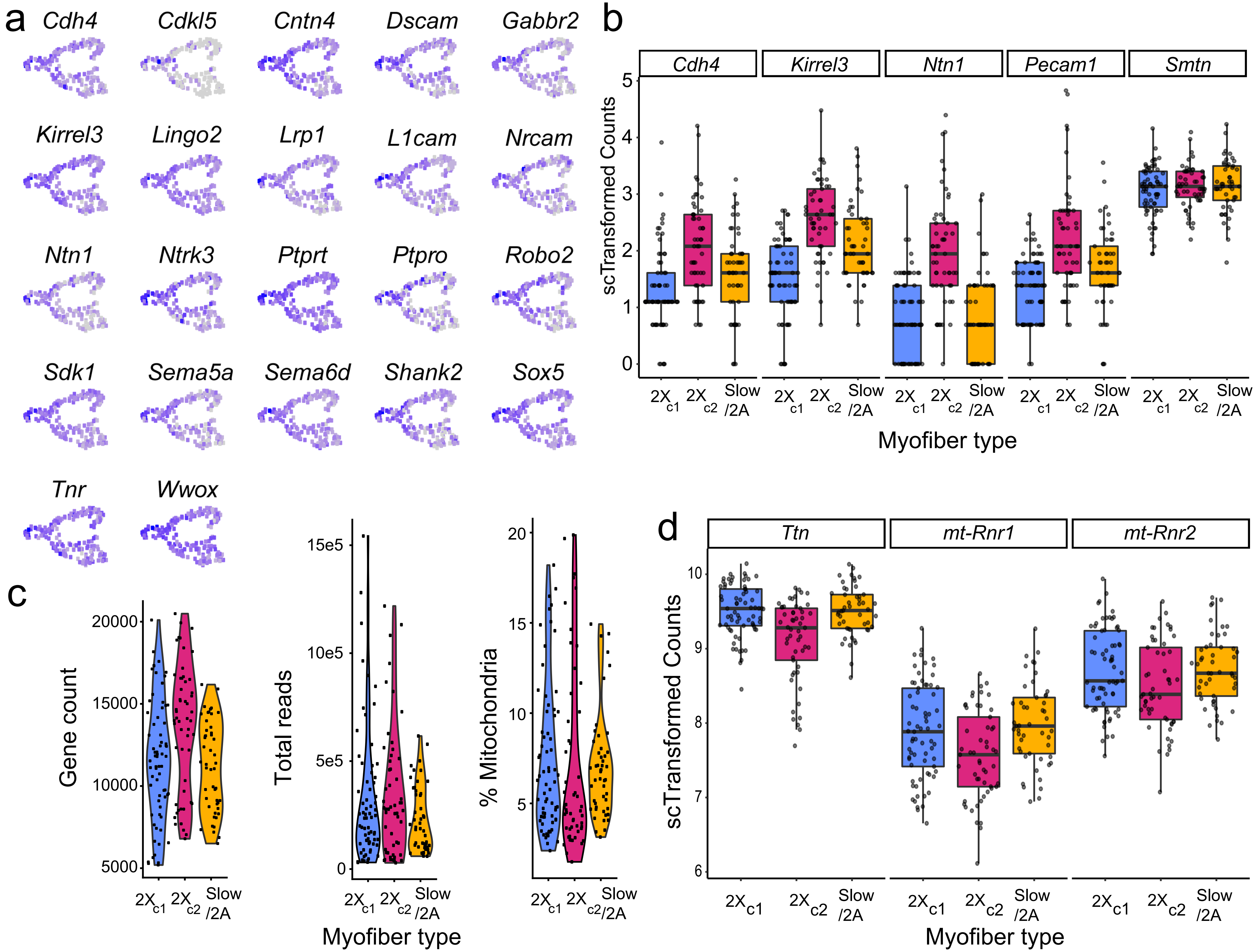

Supplementary Figure S2. Comparison of Fast 2Xc1 and Fast 2Xc2 subsets. (**a**) Twenty-two neuronal or NMJ-related genes are detected in most cells, but enriched in Fast 2Xc1 (middle left) cells. (**b**) Three neural genes (*Cdh4*, *Kirrel3*, *Ntn1*), an endothelial specific gene (*Pecam1*) and a smooth muscle cell gene (*Smtn*) are all increased in Fast 2Xc2 subsets suggesting overall increase of ambient RNA in these cells. (**c**) Total gene counts are elevated in Fast2Xc2 subsets despite no increase in total reads or % mitochondria. (**d**) Typically abundant genes, *Ttn*, *mt-Rnr1,* and *mt-Rnr2* are all of lower expression in Fast 2Xc2 cells.
